# Analysis of the association between urinary glyphosate exposure and fatty liver index: a study for US adults

**DOI:** 10.1186/s12889-024-18189-3

**Published:** 2024-03-05

**Authors:** Kexing Han, Long Gao, Honghai Xu, Jiali Li, Lianxiu Han, Jiapei Shen, Weijie Sun, Yufeng Gao

**Affiliations:** Department of Infectious Diseases, The First Affiliated Hospital of Anhui Medical University, 230022 Hefei, China

**Keywords:** Non-alcoholic fatty liver disease, Fatty liver index, Environmental exposure, Glyphosate, Cross-sectional study, NHANES

## Abstract

**Background:**

Non-alcoholic fatty liver disease (NAFLD) is a prevalent condition that often goes unrecognized in the population, and many risk factors for this disease are not well understood. Glyphosate (GLY) is one of the most commonly used herbicides worldwide, and exposure to this chemical in the environment is significant. However, studies exploring the association between GLY exposure and NAFLD remain limited. Therefore, the aim of this study was to assess the association between urinary glyphosate (uGLY) level and fatty liver index (FLI) using data from the National Health and Nutrition Examination Survey (NHANES), which includes uGLY measurements.

**Methods:**

The log function of uGLY was converted and expressed as Log_e_(uGLY) with the constant “e” as the base and used for subsequent analysis. The association between Log_e_(uGLY) (the independent variable) level and FLI (the dependent variable) was assessed by multiple linear regression analysis. Smoothing curve fitting and a generalized additive model were used to assess if there was a nonlinear association between the independent and the dependent variables. A subgroup analysis was used to find susceptible individuals of the association between the independent variable and the dependent variable.

**Results:**

A final total of 2238 participants were included in this study. Participants were categorized into two groups (< -1.011 and ≥ -1.011 ng/ml) based on the median value of Log_e_(uGLY). A total of 1125 participants had Log_e_(uGLY) levels ≥ -1.011 ng/ml and higher FLI. The result of multiple linear regression analysis showed a positive association between Log_e_(uGLY) and FLI (Beta coefficient = 2.16, 95% CI: 0.71, 3.61). Smoothing curve fitting and threshold effect analysis indicated a linear association between Log_e_(uGLY) and FLI [likelihood ratio(LLR) = 0.364]. Subgroup analyses showed that the positive association between Log_e_(uGLY) and FLI was more pronounced in participants who were female, aged between 40 and 60 years, had borderline diabetes history, and without hypertension history. In addition, participants of races/ethnicities other than (Mexican American, White and Black) were particularly sensitive to the positive association between Log_e_(uGLY) and FLI.

**Conclusions:**

A positive linear association was found between Log_e_(uGLY) level and FLI. Participants who were female, 40 to 60 years old, and of ethnic backgrounds other than Mexican American, White, and Black, deserve more attention.

**Supplementary Information:**

The online version contains supplementary material available at 10.1186/s12889-024-18189-3.

## Introduction

Non-alcoholic fatty liver disease (NAFLD) is defined as a degree of steatosis of the liver in the absence of excessive alcohol consumption and other known causes [1], and has now become one of the most common liver diseases in the world. As a hepatic manifestation of the metabolic syndrome, NAFLD has seen its prevalence rise with the global economic enhancement [2, 3]. Previous studies have shown an association between the development of NAFLD and factors such as obesity, diet, exercise, and genetic variation [4]. As the research on NAFLD gradually intensified, researchers found that environmental factors also contribute significantly to the development of NAFLD, such as air pollution [5]. However, due to the diversity of environmental factors, it is a common doubt and interest to be exposed to which harmful environmental substances might play a contributing role in the development of NAFLD.

Although the risk factors for the development of the vast majority of diseases are still unknown, residual components of pesticides in the environment has been implicated in many human health outcomes [6, 7]. Glyphosate herbicides (GBHs) were marketed in 1974 and are among the most commonly used herbicides in the world, accounting for nearly 72% of global pesticide use [8]. Previous studies have shown that glyphosate (GLY) is widely present in ecosystems, such as soil, water and indoor dust [9, 10]. Increased levels of GLY can also be detected in the food chain, which may be related to the overuse of GLY in crops [11]. In conclusion, the widespread use of GLY has increased the risk to animals and humans of exposure to residual GLY in the environment. Based on toxicity tests conducted by the patent holder of GLY (i.e., Monsanto) in the 1970 and 1980 s, GLY was described as “virtually nontoxic” to animals and humans [12]. However, additional studies have found that GLY could be toxic to multiple organs, including but not limited to nephrotoxicity [13], hepatotoxicity [14], gastrointestinal [15], cardiovascular [16], respiratory [17], and reproductive systems [18]. The liver, the second most susceptible organ to GLY, has also been demonstrated in several studies. mills PJ demonstrated a significant increase in GLY excretion in patients with steatohepatitis [19]. Mesnage R demonstrated that chronic ultra-low dose GLY exposure can lead to liver dysfunction and that the resulting proteomic and metabolomic abnormalities overlap with NAFLD [20]. However, researches on the association of GLY with NAFLD were still limited so far.

Based on previous studies, we speculated that there is an association between GLY and the development of NAFLD which we proposed to validate with samples from the NHANES program. However, since liver biopsies are difficult to obtain in the NHANES program, but fatty liver index (FLI) has been validated as a valid tool for assessing NAFLD [21, 22]. Therefore, we aimed to evaluate the association between uGLY levels and FLI with data from NHANES 2013–2016 adult participants.

## Methods

### Data source

NHANES was established by the National Center for Health Statistics (NCHS) of the Centers for Disease Control and Prevention (CDC), and contains information on questionnaires, exams, and laboratory tests for selected study participants. The data contained in NHANES is open to the public, which allowed us to be exempt from ethical review of the study [23].

### Study participants

**Fig. 1:**
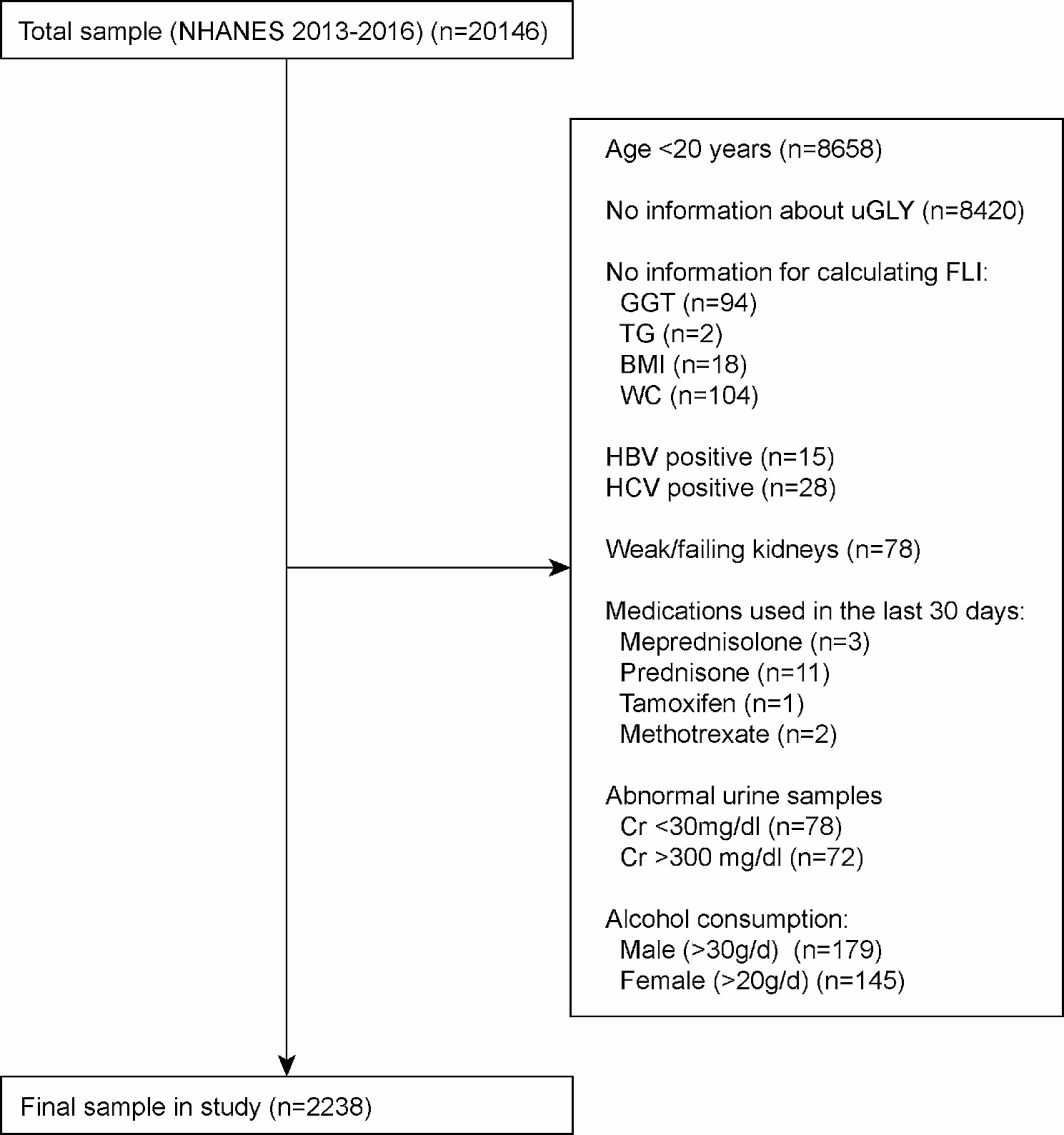
Flow chart for participants

A total of 20,146 participants were included in the NHANES 2013–2016 survey. Relying on the determination of adult age in previous NHANES-based cross-sectional studies [24–26], we first excluded participants younger than 20 years of age (*n* = 8658). Participants without clear uGLY information were then excluded (*n* = 8420), followed by those missing laboratory indicators used to calculate FLI, which were glutamyl transpeptidase (GGT) (*n* = 94), triglycerides (TG) (*n* = 2), body mass index (BMI) (*n* = 18), and waist circumference (WC) (*n* = 104). Since there is a close association between steatosis of the liver and viral hepatitis [27, 28]. Therefore, participants who were positive for hepatitis B virus (HBV) (*n* = 15) and hepatitis C virus (HCV) (*n* = 28) were also not included in this study. As urine is one of the important routes for GLY excretion out of the body [29], we excluded patients with renal weakness or renal failure (*n* = 78). Previous studies have confirmed that the side effects of glucocorticoids, tamoxifen and methotrexate lead to disturbances in fat metabolism of the liver [30–32]. Therefore, we excluded participants (*n* = 17) who had apparently used medications that interfere with fat metabolism ( in the past month). Specific drugs included meprednisolone (*n* = 3), prednisolone (*n* = 11), tamoxifen (*n* = 1) and methotrexate (*n* = 2). World Health Organization (WHO) recommends that if a urine sample is too dilute (creatinine concentration < 30 mg/dL) or too concentrated (creatinine concentration > 300 mg/dL), urine should be recollected and analyzed for creatinine and target chemicals [33]. Therefore, samples with urine that was too dilute (*n* = 78) or too concentrated (*n* = 72) were excluded from this study. In addition, participants were excluded for excessive alcohol consumption, defined as drinking more than 30 g of alcohol/day for male (*n* = 179) and more than 20 g/day for female (*n* = 145) [34]. Ultimately, a total of 2238 participants were included in this study. More details of the participants being screened were shown in Fig. 1.

### Independent and dependent variables

uGLY was defined as the independent variable. Urine samples from approximately one-third of participants aged 6 years and older were used to measure GLY concentrations (ng/ml) during the two survey cycles of NHANES 2013–2016 in total. Urine was collected at a mobile examination center (MEC) and subsequently aliquoted within a few hours and frozen in time for transport to the CDC’s National Center for Environmental Health (NCEH) for testing. A 200 µl urine sample was utilized, based on two-dimensional on-line ion chromatography with tandem mass spectrometry (IC-MS/MS) and isotope dilution quantification [35]. For analytes with analytical results below the lower limit of detection (LLOD), the analytic results were filled and placed using an estimated value, which was the LLOD divided by a square root of 2. The LLOD for uGLY was 0.2 ng/ml.

FLI was defined as the dependent variable, which had been validated as a valid tool for assessing NAFLD [21, 22]and was obtained by the following formula [36]:

Fatty Liver Index (FLI) = (e^0.953×Ln (TG)^ + 0.139×BMI + 0.718×Ln(GGT) + 0.053×WC − 15.745) ÷ (1 + e^0.953×Ln (TG)^ + 0.139×BMI + 0.718×Ln(GGT) + 0.053×WC − 15.745) × 100. TG, GGT, BMI and WC in the calculation formula represent triglycerides, glutamyl transpeptidase, body mass index and waist circumference, respectively. TG (mg/dl) and GGT (U/L) were derived from laboratory test information, and BMI (kg/m^2^) and WC (cm) were obtained from physical examination information.

### Covariates

In order to better demonstrate accurate independent effects between independent and dependent variables, we included covariates that could potentially affect uGLY and FLI score in the model for adjustment. Questionnaire information: age (years), gender, race/ethnicity, education level, ratio of family income to poverty (PIR), hypertension, diabetes, physical activity intensity, smoking, herbicide use, and fasting time (hours). PIR represents the ratio of family income to poverty, which is calculated using the Department of Health and Human Services (HHS) poverty guidelines as a measure. We also inducted information on dietary intake of some nutrients: energy (Kcal), protein (gm), sugar (gm), fat (gm), cholesterol (mg), alcohol (gm), and moisture (gm). All participants in NHANES 2013–2016 underwent two 24-hour dietary recalls, and we had the average of the two recall information for the final analysis [37, 38]. Laboratory test information was included for alanine aminotransferase (ALT) (U/L), aspartate aminotransferase (AST) (U/L), creatine phosphokinase (CPK) (U/L), albumin (ALB) (g/dl), globulin (GLB) (g/dl), total bilirubin (TBIL) (mg/dl), blood urea nitrogen (BUN) (mg/dl), uric acid (UA) (mg/dl), creatinine (Cr) (mg/dl), total cholesterol (TC) (mg/dl), and serum iron (ug/dl). Considering that inflammation is a major factor affecting FLI, but c-reactive protein (CRP) information was not included in NHANES 2013–2016, previous studies demonstrated a close association between systemic immune inflammatory index (SII) and liver disease [39, 40]. Therefore, we also calculated SII as a covariate with the information of lymphocyte count (LYM) (1000 cells/UL), neutrophil count (NEU) (1000 cells/UL) and platelet count (PLT) (1000 cells/UL), calculated as [41]: SII = NEU/LYM × PLT.

### Statistical analysis

We analyzed all data in this study using R (http://www.R-project.org) and EmpowerStats (http://www.empowerstats.com), and *p* < 0.05 was determined to be statistically significant. NHANES employs complex multi-stage probability sampling, and the sampling weights provided should be used appropriately in the statistical analysis to make the obtained sample representative. According to the official NHANES recommendations, the uGLY sample was tested only in 1/3 of the sample and unique subweights (WTSSCH2Y) were provided to obtain the final weights included in this study from the sum of the weights from NHANES 2013–2014 and NHANES 2015–2016 cycles divided by 2 [42]. Continuous variables were presented in the form of median and quartiles, and continuous variables with no more than 10% missing values could be filled with the mean, otherwise they were converted to dichotomous variables using the median as a cut-off, and the missing values were set as a separate group. Categorical variables with more missing values were set as a separate group for inclusion in the analysis. The normality test revealed that the available uGLY data were skewed. Therefore, we transformed the log function of uGLY with the constant “e” as the base [Log_e_(uGLY)] and used it for all the analyses in this study. Participants were categorized into two groups (< -1.011 ng/ml and ≥ -1.011 ng/ml) based on the median of Log_e_(uGLY) level. For the comparison of all variables between the two groups, the data set was parsed and weights were obtained by using the survey design R package as recommended on the NHANES website, and p-values were calculated with weighted linear regression (continuous variables) and chi-square tests (categorical variables). We screened the selected covariates before building the final model [43]. First, a stepwise screening based on the variance inflation factor (VIF) removed covariates with too high covariance (VIF > 5) (Supplementary Table 1). Subsequently, the final included covariates (Supplementary Table 4) were identified based on the principle that the introduction of a covariate in the basic model or its exclusion from the full model would have an effect of > 10% on the regression coefficient of Log_e_(uGLY) (Supplementary Table 2), or that the introduced covariate would have a statistical p-value < 0.1 on the regression coefficient of FLI (Supplementary Table 3). Multiple linear regression analysis was used to assess the association between Log_e_(uGLY) and FLI, and by adjusting for different covariates we generated three models. Model 1: without adjusting for any covariates, Model 2: age, gender, race/ethnicity, education level, and PIR were adjusted, and Model 3: all covariates within Supplementary Table 4 were adjusted. To further demonstrate the association between different Log_e_(uGLY) levels and FLI, we also built a model based on inverse treatment probability weighting (IPTW). Briefly, we constructed a logistic regression model with all screened covariates that could be used to determine the predicted probability of each independent participant being categorized into different Log_e_(uGLY) levels (< -1.011 ng/ml and ≥ -1.011 ng/ml). The inverse of the difference between the actual probability and the predicted probability of each participant being categorized into different groups was used as weights in subsequent model. Based on the use of weights, the differences in covariates between the different Log_e_(uGLY) groups could be balanced thereby more correctly assessing the association between different levels of Log_e_(uGLY) and FLI. It is worth noting that the independent variables in the IPTW-based model were the groups (< -1.011 ng/ml and ≥ -1.011 ng/ml) after grouping at the median of Log_e_(uGLY). Smooth curve fitting (penalized spline method) was used to evaluate whether there was a nonlinear association between Log_e_(uGLY) and FLI, and a generalized additive model was further used to assess the threshold effect between the two. Threshold effect analyses aims to find a particular threshold of the independent variable and test for inconsistency in the association between the independent variable and the response variable before and after this threshold [44]. A likelihood ratio (LLR) of less than 0.05 is generally considered a criterion for the existence of a nonlinear association. Finally, we performed subgroup analysis to identify susceptible individuals for the association between Log_e_(uGLY) and FLI and performed an interaction test.

## Results

### Baseline characteristics of participants

A total of 2238 participants were eventually enrolled in the study. Participants with Log_e_(uGLY) level ≥ -1.011 ng/ml had significantly higher FLI than those with Log_e_(uGLY) level < -1.011 ng/ml, with a statistically significant difference between the two groups (*P* < 0.001). The baseline characteristics of the participants were shown in Table 1.

**Table 1:** Characteristics of participants

Characteristic	Log_e_(uGL) (< -1.011 ng/ml)	Log_e_(uGL) (≥ -1.011 ng/ml)	P-value
Sample size	1113	1125	
Questionnaire information			
Gender (%)			< 0.001
Male	42.82	51.25	
Female	57.18	48.75	
Age (yrs)	47.00 (33.00–61.00)	50.00 (35.00–65.00)	< 0.001
Race/ethnicity (%)			0.14
Mexican American	16.17	15.11	
White	37.47	41.16	
Black	18.60	19.56	
Other Race	27.76	24.18	
Educational level (%)			0.16
Less than high school	12.36	15.12	
High school	21.03	20.60	
More than high school	66.61	64.28	
PIR (%)	2.49 (1.26–3.82)	2.20 (1.15–3.55)	0.046
Hypertension (%)			0.002
Yes	29.26	35.38	
No	70.74	64.62	
Diabetes (%)			< 0.001
Yes	6.97	12.31	
No	91.50	84.75	
Borderline	1.53	2.94	
Physical Activity(%)			0.0013
Vigorous	41.31	34.06	
Moderate	28.41	30.33	
Never	30.28	35.61	
Smoking (%)			0.19
Now	17.03	19.82	
Ever	22.71	23.08	
Never	60.25	57.10	
Used weed killer (%)			< 0.0025
Yes	5.15	8.82	
No	87.64	83.66	
Unclear	7.21	7.52	
Fasting time (hours)	10.00 (2.00–12.00)	5.00 (2.00–11.00)	< 0.001
Dietary Information			
Energy (kcal) (%)			0.15
< 1900	40.89	37.12	
≥ 1900	42.32	46.04	
Unclear	16.79	16.84	
Protein (gm) (%)			0.38
< 75.28	38.57	41.22	
≥ 75.28	44.64	41.94	
Unclear	16.79	16.84	
Sugar (gm) (%)			0.55
< 94.57	43.01	37.21	
≥ 94.57	40.20	45.95	
Unclear	16.79	16.84	
Fat (gm) (%)			0.82
< 73.68	41.06	40.00	
≥ 73.68	39.98	41.24	
Unclear	18.96	18.76	
Cholesterol (mg) (%)			0.80
< 253.50	39.98	41.33	
≥ 253.50	41.06	39.91	
Unclear	18.96	18.76	
Alcohol (gm) (%)			0.72
< 0	62.89	64.36	
≥ 0	18.15	16.89	
Unclear	18.96	18.76	
Moisture (gm) (%)			0.17
< 2541.12	38.72	42.40	
≥ 2541.12	42.32	38.84	
Unclear	18.96	18.76	
Testing Information			
ALT (U/L)	20.00 (16.00–27.00)	20.00 (16.00–28.00)	0.76
AST (U/L)	22.00 (19.00–27.00)	22.00 (19.00–27.00)	0.84
GGT (U/L)	19.00 (13.00–28.00)	19.00 (13.00–28.00)	0.95
CPK (IU/L)	105.00 (75.00-167.00)	109.00 (76.00-175.00)	0.23
ALB (g/dl)	4.30 (4.10–4.50)	4.20 (4.00-4.50)	< 0.001
GLB (g/dl)	2.80 (2.50–3.10)	2.80 (2.50–3.10)	0.66
TBIL (mg/dl)	0.60 (0.40–0.70)	0.50 (0.40–0.70)	0.01
BUN (mg/dl)	13.00 (10.00–16.00)	13.00 (11.00–17.00)	< 0.001
UA (mg/dl)	5.30 (4.30–6.30)	5.30 (4.40–6.20)	0.91
Cr (mg/dl)	0.83 (0.69–0.97)	0.86 (0.71-1.00)	< 0.001
TC (mg/dl)	191.00 (166.00-217.00)	186.00 (159.00-215.00)	0.008
TG (mg/dl)	115.00 (77.00-185.00)	123.00 (83.00-195.00)	0.017
Serum iron (ug/dl)	80.00 (61.00-104.00)	76.00 (56.00–97.00)	< 0.001
LYM (1000 cells/UL)	2.10 (1.70–2.60)	2.10 (1.70–2.60)	0.95
NEU (1000 cells/UL)	4.00 (3.10-5.00)	4.10 (3.20–5.30)	0.02
PLT (1000 cells/UL)	230.00 (198.00-272.00)	231.00 (196.00-271.00)	0.72
SII	434.00 (309.84–592.00)	450.94 (314.27-630.67)	0.06
FLI	53.28 (21.89–83.73)	62.71 (27.56–88.21)	< 0.001

Median and quartiles for continuous variables: *P*-value was calculated by weighted linear regression model

% for Categorical variables: *P*-value as calculated by chi-square test

### The association between Log_e_(uGLY) level and FLI

In all models (models 1–3), there was a positive association between Log_e_(uGLY) level and FLI. The fully adjusted model (model 3) results suggested that each 1-unit increase in Log_e_(uGLY) would be accompanied by a 2.16-unit increase in FLI (Beta coefficient = 2.16, 95% CI: 0.71, 3.61). Subsequently, we displayed Log_e_(uGLY) in tertile 1 (-1.959- -1.190), Tertile 2 (-1.191- -1.030) and Tertile 3 (-1.031- 0.104). Compared to Tertile 1, the trend of positive association between independent and dependent variables was more significant as Log_e_(uGLY) increased (P for trend < 0.001), and was most pronounced in Tertile 3 (Beta coefficient = 4.19, 95% CI: 1.49, 6.88). All results were presented in Table 2. In addition, we further validated the positive association between Log_e_(uGLY) and FLI by employing IPTW (Beta coefficient = 2.07, 95% CI: 0.18, 3.96) (Supplementary Table 6). Information on baseline characteristics of participants after IPTW was displayed in Supplementary Table 5.

**Table 2:** Association of Log_e_(uGLY) level with FLI

Characteristic	Model 1, Beta coefficient (95%CI)	Model 2, Beta coefficient (95%CI)	Model 3, Beta coefficient (95%CI)
Log_e_(uGLY)(ng/ml)	4.06 (2.29, 5.83)	2.75 (1.02, 4.48)	2.16 (0.71, 3.61)
Categories			
Tertile 1	0	0	0
Tertile 2	7.15 (3.83, 10.47)	6.71 (3.49, 9.93)	2.64 (0.01, 5.29)
Tertile 3	7.82 (4.49, 11.14)	5.53 (2.29, 8.77)	4.19 (1.49, 6.88)
P for trend	< 0.001	0.0012	< 0.001

Model 1: no covariates were adjusted; Model 2: age, gender, race/ethnicity were adjusted. Model 3: all covariates were adjusted

### Subgroup analysis of the association between Log_e_(uGLY) level and FLI

To verify the stability of the positive association between Log_e_(uGLY) level and FLI in the cohort with characteristics, we performed a subgroup analysis. In the fully adjusted model, the results of the subgroup analysis showed that this positive association were more significant in participants who were female (Beta coefficient = 2.80, 95% CI: 0.74, 4.85), 40–60 years old (Beta coefficient = 3. 80, 95% CI: 0.71, 5.44), other races/ethnicities (Beta coefficient = 5.19, 95% CI: 2.29, 8.08), without hypertension (Beta coefficient = 1.84, 95% CI: -0.52, 4.20) and borderline diabetes (Beta coefficient = 2.21, 95% CI: -8.17, 12.59). In addition, we performed interaction tests for gender, age, race/ethnicity, hypertension, and diabetes to further commit the stability of the results in the subgroup analysis (*P* > 0.05 for interaction test). The results of the subgroup analysis were presented in Table 3.

**Table 3:** Results of subgroup analysis

Characteristic	Model 1, Beta coefficient (95%CI)	Model 2, Beta coefficient (95%CI)	Model 3, Beta coefficient (95%CI)	P for interaction*
Stratified by gender				0.22
Male	1.71 (-0.74, 4.16)	0.03 (-2.39, 2.45)	0.87 (-1.16, 2.90)	
Female	5.51 (3.01, 8.01)	5.08 (2.62, 7.54)	2.80 (0.74, 4.85)	
Stratified by age (yrs)				0.22
< 40	2.94 (-0.40, 6.28)	2.72 (-0.61, 6.05)	0.62 (-1.94, 3.17)	
40–60	4.16 (1.35, 6.96)	4.32 (1.57, 7.07)	3.08 (0.71, 5.44)	
> 60	2.57 (-0.24, 5.37)	1.63 (-1.21, 4.48)	2.11 (-0.48, 4.70)	
Stratified by race/ethnicity				0.35
Mexican American	4.96 (0.40, 9.51)	3.08 (-1.46, 7.62)	0.13 (-3.75, 4.02)	
White	3.61 (0.84, 6.38)	2.13 (-0.56, 4.82)	1.81 (-0.45, 4.06)	
Black	2.99 (-1.17, 7.16)	2.49 (-1.55, 6.53)	1.48 (-1.96, 4.92)	
Other Race	6.94 (3.30, 10.58)	6.23 (2.66, 9.79)	5.19 (2.29, 8.08)	
Stratified by hypertension				0.82
Yes	3.89 (1.64, 6.14)	2.85 (0.64, 5.06)	1.71 (0.09, 3.50)	
No	1.20 (-1.26, 3.65)	1.19 (-1.24, 3.63)	1.84 (-0.52, 4.20)	
Stratified by diabetes				0.96
Yes	1.54 (-1.83, 4.91)	1.87 (-1.51, 5.25)	1.45 (-2.10, 4.99)	
No	3.11 (1.17, 5.05)	2.07 (0.18, 3.97)	2.10 (0.52, 3.69)	
Borderline	1.99 (-10.78, 14.76)	1.98 (-9.41, 13.37)	2.21 (-8.17, 12.59)	

Model 1: no covariates were adjusted; Model 2: age, gender, race/ethnicity were adjusted. Model 3: all covariates were adjusted

*In the subgroup analysis stratified by each covariate, the model is not adjusted for the stratification variable itself

The log function conversion of uGLY with the constant “e” as the base was performed and used for the analysis

### Positive linear association between Log_e_(uGLY) level and FLI

**Fig. 2:**
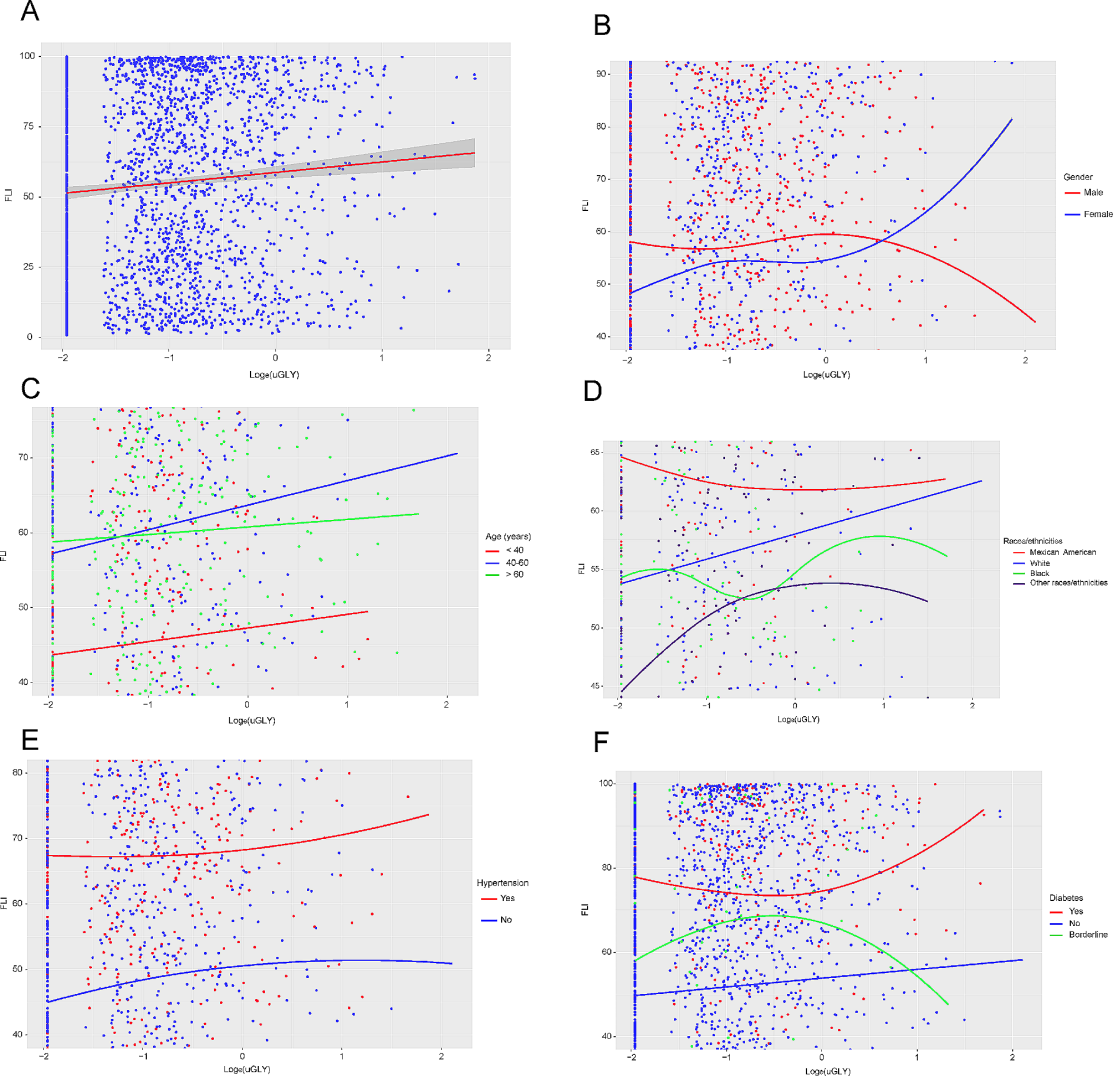
Smoothed curve fit of the association between Log_e_(uGLY) level and FLI. (**A**) The association of Log_e_(uGLY) level with FLI for all participants. The solid red line represents a smooth curve fit of the association between the independent variable and dependent variable, and the gray band represents the 95% confidence interval of the fit. Each blue point represents a sample. (**B**-**F**) The association of Log_e_(uGLY) level with FLI stratified by gender, age, race/ethnicity, hypertension and diabetes. Specific colors in each figure represents the subgroup characteristics of the participants. The lines represent a smooth curve fit between the independent variable and dependent variable. Each point represents a sample. *All the covariates were adjusted

Finally, we evaluated whether there was a nonlinear association between Log_e_(uGLY) level and FLI with smooth curve fitting and threshold effect analysis. Depending on Fig. 2A; Table 4, a linear association (LLR = 0.364) was observed between independent and dependent variables. In addition, we also designed to verify whether this positive linear association was stable in each subgroup mentioned above, for which we performed smoothed curve fitting and threshold effect analysis for each subgroup respectively. The results suggested that the positive linear association between independent and dependent variables was durable for gender (Fig. 2B and Supplementary Table 7), age (Fig. 2C and Supplementary Table 8), and hypertension (Fig. 2E and Supplementary Table 9). However, when diabetes was the subgroup, a significant increase in FLI (Beta coefficient = 5.12, 95% CI: 0.69, 9.55) would occur when the Log_e_(uGLY) of participants with diabetes exceeded − 1.55 (LLR = 0.007) (Table 5; Fig. 2F). In addition, the black race/ethnicity participants had an effect value of (Beta coefficient = 17.00, 95% CI: 2.74, 31.25) between Log_e_(uGLY) and FLI when Ln(uGLY) exceeded − 0.12 (LLR = 0.026). Participants of other races/ethnicities showed a positive association with FLI only when Log_e_(uGLY) was less than − 0.46 (Beta coefficient = 8.95, 95% CI: 4.74, 13.16) (LLR = 0.015), although when Log_e_(uGLY) was more than − 0.46 it showed a negative association with FLI which was not statistically different (Beta coefficient=-5.26, 95% CI: -14.25, 3.72) (Table 6; Fig. 2D).

**Table 4:** Threshold effect analysis of the association between Log_e_(uGLY) level and FLI

Outcome:	FLI
Linear model	
Beta coefficient (95%CI)	2.16 (0.71, 3.61)
Non-linear model	
Inflection Point(K)	-0.79
Beta coefficient (95%CI) (< K)	3.16 (0.56, 5.77)
Beta coefficient (95%CI) (> K)	0.99 (-1.92, 3.91)
LLR	0.364

All covariates were adjusted in linear model and non-linear model. The log function conversion of uGLY with the constant “e” as the base was performed and used for the analysis

**Table 5:** Threshold effect analysis of Log_e_(uGLY) level and FLI stratified by diabetes

Diabetes	Yes	No	Borderline
Linear model			
Beta coefficient (95%CI)	1.45 (-2.10, 4.99)	2.10 (0.52, 3.69)	2.21 (-8.17, 12.59)
Non-linear model			
Inflection Point(K)	-1.55	-1.33	0.07
Beta coefficient (95%CI) (< K)	-26.53 (-47.43, -5.63)	5.22 (0.09, 10.36)	11.64 (-0.88, 24.16)
Beta coefficient (95%CI) (> K)	5.12 (0.69, 9.55)	0.91 (-1.54, 3.36)	-52.56 (-98.44, -6.68)
LLR	0.007	0.209	0.008

All covariates were adjusted in linear model and non-linear model. The log function conversion of uGLY with the constant “e” as the base was performed and used for the analysis

**Table 6:** Threshold effect analysis of Log_e_(uGLY) level and FLI stratified by race

Race/ethnicity	Mexican American	White	Black	Other Race
Linear model				
Beta coefficient (95%CI)	0.13 (-3.75, 4.02)	1.81 (-0.45, 4.06)	1.48 (-1.96, 4.92)	5.19 (2.29, 8.08)
Non-linear model				
Inflection Point(K)	-0.69	0.6	-0.12	-0.46
Beta coefficient (95%CI) (< K)	-1.66 (-7.37, 4.06)	1.44 (-1.03, 3.90)	-1.57 (-5.94, 2.81)	8.95 (4.74, 13.16)
Beta coefficient (95%CI) (> K)	3.85 (-5.65, 13.35)	8.72 (-10.02, 27.46)	17.00 (2.74, 31.25)	-5.26 (-14.25, 3.72)
LLR	0.391	0.463	0.026	0.015

All covariates were adjusted in linear model and non-linear model. The log function conversion of uGLY with the constant “e” as the base was performed and used for the analysis

### Supplementary results

**Fig. 3:**
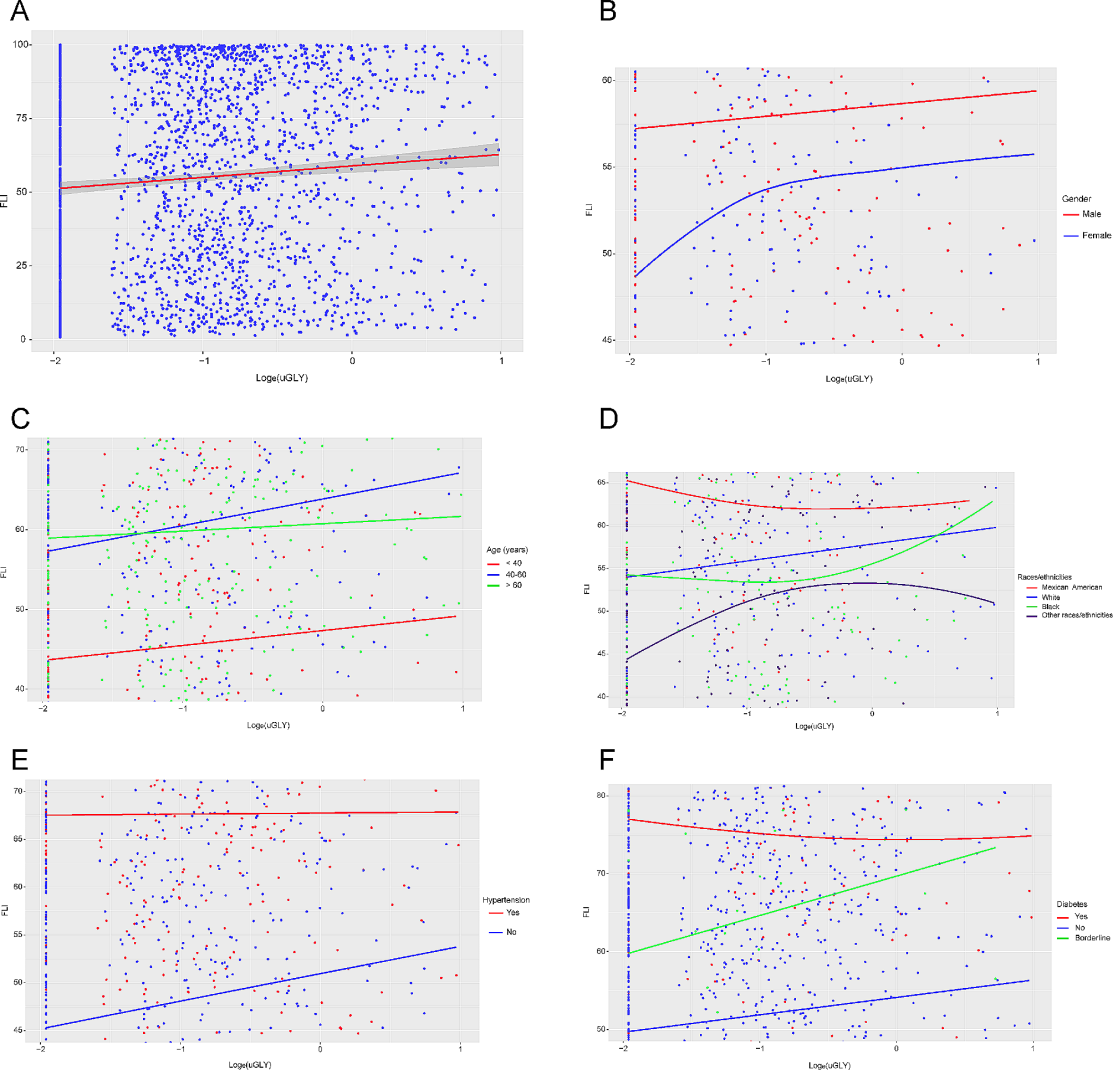
The association of Log_e_(uGLY) level with FLI after excluding outliers. (**A**) The association of Log_e_(uGLY) level with FLI for all participants. The solid red line represents a smooth curve fit of the association between the independent variable and dependent variable, and the gray band represents the 95% confidence interval of the fit. Each blue point represents a sample. (**B**-**F**) The association of Log_e_(uGLY) level with FLI stratified by gender, age, race/ethnicity, hypertension and diabetes. Specific colors in each figure represents the subgroup characteristics of the participants. The lines represent a smooth curve fit between the independent variable and dependent variable. Each point represents a sample. *All the covariates were adjusted

We excluded participants who did not have information about uGLY (*n* = 15,408) and FLI (*n* = 1286) explicitly, while retaining participants who were younger than 20 years of age, consumed large amounts of alcohol, were taking medications that interfere with fat metabolism, had viral hepatitis, had substandard urine samples, and were in renal weakness/failure to perform sensitivity analyses for exclusion criteria on the association between Log_e_(uGLY) and FLI (Supplementary Fig. 1). The results demonstrated that the true association between Log_e_(uGLY) and FLI would be severely affected when participants in Fig. 1 were not excluded (Beta coefficient = 0.01, 95% CI: -0.04, 0.05). The results of the sensitivity analysis to the exclusion criteria were displayed in Supplementary Table 10. In the validation results of the positive linear association between Log_e_(uGLY) level and FLI, through Fig. 2A we found that there were outliers in Log_e_(uGLY), for which we examined the data distribution of Log_e_(uGLY) (Supplementary Table 11). Through the results we could observe that the sample size of Log_e_(uGLY) over 1 is 26, which accounted for 1.162% of the total sample size, such a result belonged to small sample events (< 5%). For this reason, we removed these outliers and re-examined the association between Log_e_(uGLY) level and FLI. Based on the results, we found that after removing the outliers, the supplemental results were absolutely consistent with our results above. The supplemental results were shown in Supplementary Tables 12–20, Fig. 3A-F.

## Discussion

The association between environmental exposure to chemical factors and the incidence of NAFLD is a public health problem of global concern. Our study investigated the association between Log_e_(uGLY) level and FLI in US adults using nationally representative data. After adjusting for relevant covariates such as sociodemographic variables, diet, lifestyle, and physical activity information, we found a positive linear association between increased level of GLY in urine and the FLI, providing important new evidence for the epidemiology of NAFLD.

GLY has become a best seller worldwide since its release for sale as the most prominent ingredient of herbicides, and GLY is more likely to enter humans through the dermal, oral, and pulmonary routes [12]. Previous studies have demonstrated that even within the low dose range considered safe, impairment of liver function was observed in rats chronically exposed (2 years) to GLY [45]. The results of transcriptome sequencing indicate that gene expression profiles were dominated by lipid deposition and mitochondrial membrane dysfunction [45]. On the basis of the “two-hit” theory, mitochondrial membrane dysfunction has been proposed to be critical in the development of NAFLD, such as impaired fatty acid oxidation and oxidative phosphorylation [46]. The current theory suggested that an equally useful conceptual framework was that the liver’s ability to process major metabolic energy substrates (carbohydrates and fatty acids) was overwhelmed, leading to the accumulation of toxic lipids and thus activating the pathway of NAFLD development [47]. In another study, the investigators found that in the livers of GLY-administered rats, a trend towards accumulation of most fatty acids, particularly acylcarnitine, and a statistically significant increase in cholesterol levels were observed, and proteomics profiling further confirmed the significant changes in the metabolic processes of lipid detoxification [20].

As a new epidemiological report, we not only found that uGLY level was associated with an increase in FLI, but in order to get an adapted cohort, we further did a subgroup analysis. The most important result suggested that the positive association between uGLY and FLI was more significant in female participants compared to males. This was an interesting finding, as in a clinical observational study, the investigators found significantly higher GLY residues in the urine of women than men [19]. In addition, Mesnage R demonstrated the effects of GLY exposure on the liver by using female rats in their study [20], and in their another study, Mesnage R’s explanation for why male rats were dropped as subjects was because male animals suffered more severe liver and kidney damage than females, leading to increased premature mortality [45]. However, this would not clarify the conclusions we have obtained so far. Giommi C conducted a transcriptomic analysis using GLY-exposed zebrafish livers and showed that transcript levels of heat shock protein 70.2 were elevated in female zebrafish and decreased in male zebrafish [48]. In contrast, Antunes AM showed a sex-dependent increase in hepatocyte vascular area, with higher values in males compared to GLY-exposed female peacock fish [49]. In the epidemiology of NAFLD, previous studies have shown a higher prevalence and severity of NAFLD in males of reproductive age than in females. However, after menopause, the prevalence of NAFLD in women would exceed that in men, suggesting a protective effect of estrogen on the development of NAFLD [50]. In a cross-sectional study using the NHANES program, Geier DA observed a significant negative association between the concentration of GLY and total estradiol, as well as a negative trend between the concentration of GLY and total testosterone [42]. In conclusion, there was still no valid evidence for the effect of GLY on hepatic steatosis by gender, and our study provides a new and important evidence. In addition, we found another valid subgroup group to be participants aged 40–60 years. Based on the NHANES III survey, 16.1% of NAFLD patients were 30 to 40 years old, followed by 22.3% of those 41 to 50 years old, 29.3% of those 51 to 60 years old, and 27.6% of those over 60 years old [51]. Current studies generally supported a negative association between GLY levels and age [52–55]. In contrast, another NHANES survey found a positive association between GLY levels and age when age exceeded 20 years [42]. Thus, previous studies provided evidence for our current results. According to the most recent NHANES report, non-Hispanic whites had higher GLY exposure than other populations [55], but our results suggested that the positive association between GLY and FLI was more significant in other other races/ethnicities. The potential association was unknown to us because of the overly broad range of participant other races/ethnicities included in this subgroup. However, based on previous NHANES findings, it was similarly concluded that whites had the highest prevalence of NAFLD, while blacks had the lowest, and other other races/ethnicities were in the middle of the prevalence range [56]. For the current findings, we speculated the existence of other potential influences, such as the finding in an NHANES survey that populations classified as other other races/ethnicities would have higher dietary fiber intake compared to non-Hispanic whites [57], where grains can provide sufficient dietary fiber to be acceptable. Furthermore, we also hope that future multicenter prospective cohort studies with large samples would validate the current findings.

As the first cross-sectional study to investigate the association between GLY level and FLI, our study contains the following strengths. NHANES follows a rigorous and well-designed study protocol with a wealth of high-quality data, including measurements of many environmental contaminants of potential public health importance.The NHANES program takes into account sample weighting issues so that the results of the study obtained are broadly applicable to the general US population. Also, the sample size of our study was large enough to allow for relevant subgroup analyses that were utilized to validate the robustness of the results. However, there were still some limitations to our study. First, the cross-sectional study was unable to identify causality. Therefore, the existence of reverse causality between uGLY and FLI has yet to be verified. Second, the potential factors that might influence either FLI or uGLY level are diverse and variable, although we collected as many possible confounders as possible in NHANES and adjusted for them in the model. However, there continued to be no guarantee that other confounding factors existed that could have biased the results. Finally, the risk of population exposure to GLY and the ability of the body to metabolise it are movable, and it is still worth exploring whether the long-term effects of GLY on the human body can be captured by only one measurement. However, given the limited evidence from current studies on GLY and liver disease, we believe that the current results would still be of good value.

## Conclusions

We contributed a new evidence for the management of NAFLD by analysing data from NHANES 2013–2016. Overall, there was a positive association between Log_e_(uGLY) level and FLI, and this positive association was linear. US adults who are female, other other races/ethnicities, and aged 40–60 years should be more cautious about the higher risk of FLI associated with exposure to GLY.

### Electronic supplementary material

Below is the link to the electronic supplementary material.

Supplementary Material 1

Supplementary Material 2

## Data Availability

The datasets generated during the current study are available in database (https://www.cdc.gov/nchs/nhanes/).
