# Supplementary material for: Analysis of the association between urinary glyphosate exposure and fatty liver index: a study for US adults
Source: BMC Public Health. 2024 Mar 5;24:703. doi: 10.1186/s12889-024-18189-3 (PMC10916137; doi:10.1186/s12889-024-18189-3)
Supplement: Supplementary file 1 — Supplementary Material 1 [file 12889_2024_18189_MOESM1_ESM.doc]

Supplementary Tables:

Supplementary Table 1. Diagnosis of co-linearity of variables

| **Variables** | **Step 1** | **Step 2** | **Step 3** |
| --- | --- | --- | --- |
|  | VIF 1 | VIF 2 | VIF 3 |
| Log_e_(uGLY) (ng/ml) | 1.1 | 1.1 | 1.1 |
| Gender | 1.9 | 1.9 | 1.9 |
| Age (yrs) | 1.7 | 1.7 | 1.7 |
| Race/ethnicity | 1.1 | 1.1 | 1.1 |
| Educational level | 1.2 | 1.2 | 1.2 |
| PIR (%) | 1.3 | 1.3 | 1.3 |
| ALB (g/dl) | 221.2 | 1.4 | 1.4 |
| AST (U/L) | 2.4 | 2.4 | 2.4 |
| ALT (U/L) | 2.5 | 2.5 | 2.5 |
| BUN (mg/dl) | 1.5 | 1.5 | 1.5 |
| TC (mg/dl) | 1.1 | 1.1 | 1.1 |
| CPK (IU/L) | 1.2 | 1.2 | 1.2 |
| Cr (mg/dl) | 2 | 2 | 2 |
| GLB (g/dl) | 339.3 | 1.3 | 1.3 |
| Serum iron (ug/dl) | 1.4 | 1.4 | 1.4 |
| TBIL (mg/dl) | 1.3 | 1.3 | 1.3 |
| UA (mg/dl) | 1.6 | 1.6 | 1.6 |
| Hypertension | 1.4 | 1.4 | 1.4 |
| SII | 1.1 | 1.1 | 1.1 |
| Diabetes | 1.1 | 1.1 | 1.1 |
| Energy (kcal) | 6.1 | 6.1 | NA |
| Protein (gm) | 391.5 | NA | NA |
| Sugar (gm) | 3 | 3 | 2.5 |
| Fat (gm) | 4.7 | 4.7 | 3.1 |
| Cholesterol (mg) | 2.9 | 2.9 | 2.9 |
| Alcohol (mg) | 2.7 | 2.7 | 2.6 |
| Moisture (gm) | 2.5 | 2.5 | 2.4 |
| Fasting time (hours) | 3.5 | 3.5 | 3.5 |
| Physical Activity | 3.5 | 3.5 | 3.5 |
| Used weed killer | 1 | 1 | 1 |
| Smoking | 1 | 1 | 1 |

*Excluded variables: Energy (kcal), Protein (gm)

Supplementary Table 2. Covariates Screening ( Standard 1)

| **Covariates** | **Regression coefficient** | | **Selected** |
| --- | --- | --- | --- |
|  | **Basic Model** | **Complete Model** |  |
| Gender | 3.7161 | 2.3650 * | Yes |
| Age (yrs) | 3.1294 * | 2.0930 | Yes |
| Race/ethnicity | 4.1193 | 1.9617 |  |
| Educational level | 4.0052 | 1.9603 |  |
| PIR | 3.9614 | 2.0403 |  |
| ALB (g/dl) | 3.0740 * | 2.5640 * | Yes |
| AST (U/L) | 3.5655 * | 1.5138 * | Yes |
| ALT (U/L) | 3.5775 * | 1.6919 * | Yes |
| BUN (mg/dl) | 3.8925 | 1.6018 * | Yes |
| TC (mg/dl) | 4.5630 * | 1.7255 * | Yes |
| CPK (IU/L) | 4.0584 | 1.9474 |  |
| Cr (mg/dl) | 3.7722 | 1.9511 |  |
| GLB (g/dl) | 4.0287 | 1.9317 |  |
| Serum iron (ug/dl) | 3.4785 * | 2.1277 | Yes |
| TBIL (mg/dl) | 3.7189 | 2.0458 |  |
| UA (mg/dl) | 4.3535 | 1.0016 * | Yes |
| Hypertension | 2.9504 * | 1.9762 | Yes |
| SII | 3.8709 | 1.9833 |  |
| Diabetes | 2.9131 * | 2.2703 * | Yes |
| Sugar (gm) | 4.0947 | 1.8753 |  |
| Fat (gm) | 4.0803 | 1.9530 |  |
| Cholesterol (mg) | 4.1318 | 1.8814 |  |
| Alcohol (mg) | 4.0265 | 1.9003 |  |
| Moisture (gm) | 4.2860 | 1.8105 |  |
| Fasting time (hours) | 3.8685 | 2.0306 |  |
| Physical Activity | 3.9402 | 1.9677 |  |
| Used weed killer | 3.8878 | 2.1210 |  |
| Smoking | 4.0223 | 1.9761 |  |

The log function conversion of uGLY with the constant "e" as the base was performed and used for the analysis.

Supplementary Table 3. Covariates Screening ( Standard 2)

| **Covariates** | **β** | **SE** | **95%CI Low** | **95%CI Upp** | **P value** |
| --- | --- | --- | --- | --- | --- |
| Gender | | | | | |
| Male | Reference | | | | |
| Female | -9.0861 | 1.3883 | -11.8071 | -6.3651 | <0.001 |
| Age (yrs) | 0.4071 | 0.0397 | 0.3293 | 0.4849 | <0.001 |
| Race/ethnicity | | | | | |
| Mexican American | Reference | | | | |
| White | -6.9835 | 2.4769 | -11.8382 | -2.1288 | 0.0049 |
| Black | -8.5333 | 3.1207 | -14.6499 | -2.4167 | 0.0063 |
| Other Race | -10.3588 | 2.9054 | -16.0534 | -4.6643 | <0.001 |
| Educational level | | | | | |
| Less than high school | Reference | | | | |
| High school | -2.4773 | 2.4305 | -7.2411 | 2.2865 | 0.3082 |
| More than high school | -4.3183 | 2.0755 | -8.3862 | -0.2504 | 0.0376 |
| PIR | -1.0898 | 0.4427 | -1.9576 | -0.2220 | 0.0139 |
| ALB (g/dl) | -24.5055 | 2.0058 | -28.4368 | -20.5742 | <0.001 |
| AST (U/L) | 0.3072 | 0.0462 | 0.2166 | 0.3977 | <0.001 |
| ALT (U/L) | 0.6007 | 0.0389 | 0.5245 | 0.6768 | <0.001 |
| BUN (mg/dl) | 0.2933 | 0.1399 | 0.0192 | 0.5675 | 0.0361 |
| TC (mg/dl) | 0.1049 | 0.0158 | 0.0740 | 0.1359 | <0.001 |
| CPK (IU/L) | 0.0002 | 0.0035 | -0.0067 | 0.0072 | 0.9456 |
| Cr (mg/dl) | 13.8102 | 3.2394 | 7.4610 | 20.1594 | <0.001 |
| GLB (g/dl) | 12.6181 | 1.6497 | 9.3847 | 15.8516 | <0.001 |
| Serum iron (ug/dl) | -0.1305 | 0.0197 | -0.1690 | -0.0919 | <0.001 |
| TBIL (mg/dl) | -14.3101 | 2.2625 | -18.7447 | -9.8756 | <0.001 |
| UA (mg/dl) | 8.8147 | 0.4762 | 7.8813 | 9.7481 | <0.001 |
| Hypertension | | | | | |
| Yes | Reference | | | | |
| No | -21.6932 | 1.4242 | -24.4846 | -18.9019 | <0.001 |
| SII | 0.0087 | 0.0025 | 0.0038 | 0.0135 | <0.001 |
| Diabetes | | | | | |
| Yes | Reference | | | | |
| No | -26.3883 | 2.3107 | -30.9173 | -21.8594 | <0.001 |
| Borderline | -14.8997 | 5.0560 | -24.8094 | -4.9900 | 0.0032 |
| Sugar (gm) | | | | | |
| < 94.57 | Reference | | | | |
| ≥ 94.57 | -0.4016 | 1.5340 | -3.4082 | 2.6049 | 0.7935 |
| Unclear | -3.5004 | 2.0306 | -7.4804 | 0.4796 | 0.0849 |
| Fat (gm) | | | | | |
| < 73.68 |  | | | | |
| ≥ 73.68 | 2.2773 | 1.5386 | -0.7384 | 5.2930 | 0.1390 |
| Unclear | -2.0510 | 2.0481 | -6.0652 | 1.9632 | 0.3167 |
| Cholesterol (mg) | | | | | |
| < 253.50 | Reference | | | | |
| ≥ 253.50 | 3.0913 | 1.5326 | 0.0874 | 6.0951 | 0.0438 |
| Unclear | -1.8002 | 2.0088 | -5.7375 | 2.1370 | 0.3703 |
| Alcohol (mg) | | | | | |
| < 0 | Reference | | | | |
| ≥ 0 | -7.3714 | 1.7899 | -10.8797 | -3.8631 | <0.001 |
| Unclear | -5.0563 | 1.9108 | -8.8015 | -1.3111 | 0.0082 |
| Moisture (gm) | | | | | |
| < 2541.12 | Reference | | | | |
| ≥ 2541.12 | 8.1872 | 1.5325 | 5.1834 | 11.1910 | <0.001 |
| Unclear | 1.2481 | 2.0427 | -2.7555 | 5.2518 | 0.5412 |
| Fasting time (hours) | -3.5319 | 1.3989 | -6.2737 | -0.7901 | 0.0116 |
| Physical activity | | | | | |
| Vigorous | Reference | | | | |
| Moderate | 3.3790 | 1.7198 | 0.0082 | 6.7497 | 0.0496 |
| Never | 2.9258 | 1.6668 | -0.3412 | 6.1928 | 0.0793 |
| Used weed killer | | | | | |
| Yes | Reference | | | | |
| No | -6.2158 | 2.7539 | -11.6135 | -0.8181 | 0.0241 |
| Unclear | -0.8149 | 3.6928 | -8.0528 | 6.4231 | 0.8254 |
| Smoking | | | | | |
| Now | Reference | | | | |
| Ever | -3.0462 | 2.1891 | -7.3369 | 1.2445 | 0.1642 |
| Never | -2.8480 | 1.8680 | -6.5092 | 0.8132 | 0.1275 |

The log function conversion of uGLY with the constant "e" as the base was performed and used for the analysis.

Supplementary Table 4. The final included covariates

| **Questionnaire information** | | | | |
| --- | --- | --- | --- | --- |
| Gender | Age | Race/ethnicity | Educational level | PIR |
| Hypertension | Diabetes | Fasting time | Physical activity | Used weed killer |
| **Dietary information** | | | | |
| Sugar | Cholesterol | Alcohol | Moisture |  |
| **Testing information** | | | | |
| ALB | AST | ALT | BNU | TC |
| Cr | GLB | Serum iron | TBIL | UA |
| SII |  |  |  |  |

Supplementary Table 5. Characteristics of participants after IPTW

| **Characteristic** | **Log_e_(uGLY) (< -1.011 ng/ml)** | **Log_e_(uGLY) (≥ -1.011 ng/ml)** | **P-value** |
| --- | --- | --- | --- |
| Sample size | 1113 | 1125 |  |
| Gender (%) |  |  | 0.97 |
| Male | 47.3 | 47.4 |  |
| Female | 52.7 | 52.6 |  |
| Age (yrs) | 48.62 ± 17.18 | 48.62 ± 17.82 | 099 |
| Race/ethnicity (%) |  |  | 0.99 |
| Mexican American | 0.159 | 0.158 |  |
| White | 0.389 | 0.390 |  |
| Black | 0.188 | 0.187 |  |
| Other Race | 0.264 | 0.265 |  |
| Educational level (%) |  |  | 0.98 |
| Less than high school | 0.211 | 0.209 |  |
| High school | 0.220 | 0.219 |  |
| More than high school | 0.569 | 0.571 |  |
| PIR (%) | 2.47 ± 1.51 | 2.48 ± 1.53 | 0.90 |
| Hypertension (%) |  |  | 0.95 |
| Yes | 0.359 | 0.360 |  |
| No | 0.641 | 0.640 |  |
| Diabetes (%) |  |  | 0.99 |
| Yes | 0.126 | 0.126 |  |
| No | 0.850 | 0.850 |  |
| Borderline | 0.024 | 0.024 |  |
| Physical Activity(%) |  |  | 0.99 |
| Vigorous | 0.370 | 0.371 |  |
| Moderate | 0.311 | 0.312 |  |
| Never | 0.319 | 0.317 |  |
| Used weed killer (%) |  |  | 0.99 |
| Yes | 0.066 | 0.065 |  |
| No | 0.866 | 0.867 |  |
| Unclear | 0.068 | 0.068 |  |
| Sugar (gm) (%) |  |  | 0.97 |
| < 94.57 | 0.401 | 0.406 |  |
| ≥ 94.57 | 0.408 | 0.405 |  |
| Unclear | 0.191 | 0.189 |  |
| Cholesterol (mg) (%) |  |  | 0.99 |
| < 253.50 | 0.405 | 0.405 |  |
| ≥ 253.50 | 0.404 | 0.406 |  |
| Unclear | 0.191 | 0.189 |  |
| Alcohol (mg) (%) |  |  | 0.99 |
| < 0 | 0.633 | 0.635 |  |
| ≥ 0 | 0.176 | 0.176 |  |
| Unclear | 0.191 | 0.189 |  |
| Moisture (gm) (%) |  |  | 0.98 |
| < 2541.12 | 0.405 | 0.404 |  |
| ≥ 2541.12 | 0.404 | 0.407 |  |
| Unclear | 0.191 | 0.189 |  |
| ALT (U/L) | 24.38 ± 13.72 | 24.49 ± 19.11 | 0.87 |
| AST (U/L) | 24.92 ± 12.69 | 24.98 ± 15.86 | 0.91 |
| ALB (g/dl) | 4.27 ± 0.35 | 4.27 ± 0.34 | 0.98 |
| GLB (g/dl) | 2.84 ± 0.43 | 2.84 ± 0.43 | 0.88 |
| TBIL (mg/dl) | 0.60 ± 0.30 | 0.60 ± 0.30 | 0.87 |
| BUN (mg/dl) | 13.80 ± 5.02 | 13.84 ± 5.34 | 0.88 |
| UA (mg/dl) | 5.40 ± 1.44 | 5.40 ± 1.41 | 0.99 |
| Cr (mg/dl) | 0.87 ± 0.24 | 0.87 ± 0.23 | 0.86 |
| TC (mg/dl) | 191.27 ± 40.84 | 191.27 ± 43.74 | 0.99 |
| Serum iron (ug/dl) | 81.38 ± 33.33 | 81.48 ± 34.52 | 0.94 |
| SII | 507.21 ± 390.02 | 503.47 ± 279.10 | 0.79 |

Mean ± SD for continuous variables: *P*-value was calculated by weighted linear regression model.

% for Categorical variables: *P*-value as calculated by chi-square test.

Supplementary Table 6. Association between different Log_e_(uGLY) levels and FLI based on IPTW

| **Characteristic** | **Beta coefficient (95%CI)** |
| --- | --- |
| Log_e_(uGLY) (ng/ml) |  |
| < -1.011 ng/ml | 1.0 |
| ≥ -1.011 ng/ml | 2.07 (0.18, 3.96) |

Supplementary Table 7. Threshold effect analysis of Log_e_(uGLY) level and FLI stratified by gender

| **Gender** | **Male** | **Female** |
| --- | --- | --- |
| Linear model |  |  |
| Beta coefficient (95%CI) | 0.87 (-1.16, 2.90) | 2.80 (0.74, 4.85) |
| Non-linear model |  |  |
| Inflection Point(K) | 0.11 | 0.31 |
| Beta coefficient (95%CI) (< K) | 2.18 (-0.23, 4.58) | 1.60 (-0.79, 3.99) |
| Beta coefficient (95%CI) (> K) | -9.24 (-19.52, 1.04) | 13.98 (2.38, 25.57) |
| LLR | 0.048 | 0.054 |

All covariates were adjusted in linear model and non-linear model.

The log function conversion of uGLY with the constant "e" as the base was performed and used for the analysis.

Supplementary Table 8. Threshold effect analysis of Log_e_(uGLY) level and FLI stratified by age

| **Age (yrs)** | **< 40** | **40-60** | **> 60** |
| --- | --- | --- | --- |
| Linear model |  |  |  |
| Beta coefficient (95%CI) | 0.62 (-1.94, 3.17) | 3.08 (0.71, 5.44) | 2.11 (-0.48, 4.70) |
| Non-linear model |  |  |  |
| Inflection Point(K) | -1.32 | -1.61 | -0.6 |
| Beta coefficient (95%CI) (< K) | 6.71 (-1.05, 14.48) | 11.67 (-3.14, 26.48) | 5.21 (0.94, 9.49) |
| Beta coefficient (95%CI) (> K) | -2.10 (-6.25, 2.05) | 1.75 (-1.51, 5.02) | -2.45 (-8.07, 3.17) |
| LLR | 0.100 | 0.245 | 0.071 |

All covariates were adjusted in linear model and non-linear model.

The log function conversion of uGLY with the constant "e" as the base was performed and used for the analysis.

Supplementary Table 9. Threshold effect analysis of Log_e_(uGLY) level and FLI stratified by hypertension

| **Hypertension** | **Yes** | **No** |
| --- | --- | --- |
| Linear model |  |  |
| Beta coefficient (95%CI) | 1.71 (-0.09, 3.50) | 1.84 (-0.52, 4.20) |
| Non-linear model |  |  |
| Inflection Point(K) | 0.48 | -0.6 |
| Beta coefficient (95%CI) (< K) | 0.59 (-2.11, 3.29) | 3.83 (1.03, 6.63) |
| Beta coefficient (95%CI) (> K) | 14.51 (0.86, 28.16) | -2.35 (-6.82, 2.13) |
| LLR | 0.063 | 0.052 |

All covariates were adjusted in linear model and non-linear model.

The log function conversion of uGLY with the constant "e" as the base was performed and used for the analysis.

Supplementary Table 10. Log_e_(uGLY) association with FLI (includes participants younger than 20 years of age, presence of heavy alcohol consumption, use of medications that interfere with fat metabolism, viral hepatitis, substandard urine samples, and presence of renal weakness/failure)

| **Characteristic** | **Model 1, Beta coefficient (95%CI)** | **Model 2, Beta coefficient (95%CI)** | **Model 3, Beta coefficient (95%CI)** |
| --- | --- | --- | --- |
| Log_e_(uGLY)(ng/ml) | 0.02 (-0.02, 0.06) | 0.02 (-0.03, 0.06) | 0.01 (-0.04, 0.05) |

Model 1: no covariates were adjusted; Model 2: age, gender, race/ethnicity were adjusted. Model 3: all covariates were adjusted.

Supplementary Table 11. Distribution of Log_e_(uGLY)

| **Grouping interval lower limit** | **Grouping interval upper limit** | **Grouping interval median** | **Frequency within group** | **Percentage (%)** |
| --- | --- | --- | --- | --- |
| -2 | -1.5 | -1.75 | 597 | 26.6756 |
| -1.5 | -1 | -1.25 | 534 | 23.8606 |
| -1 | -0.5 | -0.75 | 549 | 24.5308 |
| -0.5 | 0 | -0.25 | 313 | 13.9857 |
| 0 | 0.5 | 0.25 | 146 | 6.5237 |
| 0.5 | 1 | 0.75 | 73 | 3.2618 |
| 1 | 1.5 | 1.25 | 19 | 0.8490 |
| 1.5 | 2 | 1.75 | 6 | 0.2681 |
| 2 | 2.5 | 2.25 | 1 | 0.0447 |

Supplementary Table 12. Association of Log_e_(uGLY) level with FLI after excluding outliers

| **Characteristic** | **Model 1, Beta coefficient (95%CI)** | **Model 2, Beta coefficient (95%CI)** | **Model 3, Beta coefficient (95%CI)** |
| --- | --- | --- | --- |
| Log_e_(uGLY)(ng/ml) | 4.24 (2.36, 6.11) | 2.82 (0.99, 4.65) | 2.02 (0.51, 3.53) |
| Categories |  |  |  |
| Tertile 1 | 0 | 0 | 0 |
| Tertile 2 | 7.15 (3.83, 10.47) | 6.70 (3.48, 9.92) | 2.62 (0.02, 5.26) |
| Tertile 3 | 7.73 (4.38, 11.09) | 5.40 (2.13, 8.67) | 4.04 (1.34, 6.74) |
| P for trend | < 0.001 | < 0.0084 | < 0.001 |

Model 1: no covariates were adjusted; Model 2: age, gender, race/ethnicity were adjusted. Model 3: all covariates were adjusted.

Supplementary Table 13. Association between different Log_e_(uGLY) level and FLI based on IPTW (after excluding outliers)

| **Characteristic** | **Beta coefficient (95%CI)** |
| --- | --- |
| Log_e_(uGLY) (ng/ml) |  |
| < -1.011 ng/ml | 1.0 |
| ≥ -1.011 ng/ml | 1.86 (0.13, 3.85) |

Supplementary Table 14. Results of subgroup analysis after excluding outliers

| **Characteristic** | **Model 1, Beta coefficient (95%CI)** | **Model 2, Beta coefficient (95%CI)** | **Model 3, Beta coefficient (95%CI)** | **P for interaction*** |
| --- | --- | --- | --- | --- |
| Stratified by gender |  |  |  | 0.65 |
| Male | 2.27 (-0.27, 4.81) | 0.56 (-1.93, 3.06) | 1.40 (-0.70, 3.49) |  |
| Female | 5.23 (2.52, 7.94) | 4.91 (2.25, 7.58) | 2.31 (0.14, 4.48) |  |
| Stratified by age (yrs) |  |  |  | 0.23 |
| < 40 | 3.04 (-0.36, 6.45) | 2.82 (-0.57, 6.22) | 0.65 (-1.95, 3.26) |  |
| 40-60 | 4.83 (1.74, 7.91) | 4.62 (1.59, 7.64) | 3.41 (0.89, 5.93) |  |
| > 60 | 2.78 (-0.16, 5.73) | 1.76 (-1.22, 4.74) | 2.18 (-0.53, 4.89) |  |
| Stratified by race/ethnicity |  |  |  | 0.32 |
| Mexican American | 6.15 (1.33, 10.97) | 4.34 (-0.44, 9.12) | 0.32 (-3.79, 4.43) |  |
| White | 3.65 (0.69, 6.61) | 1.81 (-1.06, 4.69) | 1.61 (-0.75, 3.97) |  |
| Black | 3.16 (-1.10, 7.42) | 2.79 (-1.33, 6.91) | 1.78 (-1.73, 5.28) |  |
| Other Race | 7.25 (3.46, 11.04) | 6.72 (3.02, 10.42) | 5.50 (2.48, 8.52) |  |
| Stratified by hypertension |  |  |  | 0.33 |
| Yes | 0.43 (-2.27, 3.14) | 0.22 (-2.46, 2.91) | 0.83 (-1.71, 3.37) |  |
| No | 4.65 (2.31, 6.98) | 3.56 (1.27, 5.84) | 2.21 (0.36, 4.06) |  |
| Stratified by diabetes |  |  |  | 0.72 |
| Yes | 0.64 (-3.01, 4.28) | 0.81 (-2.86, 4.49) | 0.28 (-3.53, 4.09) |  |
| No | 3.38 (1.34, 5.43) | 2.26 (0.26, 4.26) | 2.08 (0.43, 3.74) |  |
| Borderline | 3.83 (-9.55, 17.21) | 4.59 (-7.29, 16.47) | 4.85 (-5.75, 15.44) |  |

Model 1: no covariates were adjusted; Model 2: age, gender, race/ethnicity were adjusted. Model 3: all covariates were adjusted.

*In the subgroup analysis stratified by each covariate, the model is not adjusted for the stratification variable itself.

The log function conversion of uGLY with the constant "e" as the base was performed and used for the analysis.

Supplementary Table 15. Threshold effect analysis of the association between Log_e_(uGLY) level and FLI after excluding outliers

| **Outcome:** | **FLI** |
| --- | --- |
| Linear model |  |
| Beta coefficient (95%CI) | 2.02 (0.51, 3.53) |
| Non-linear model |  |
| Inflection Point(K) | -0.65 |
| Beta coefficient (95%CI) (< K) | 3.21 (0.81, 5.61) |
| Beta coefficient (95%CI) (> K) | -0.17 (-3.94, 3.59) |
| LLR | 0.210 |

All covariates were adjusted in linear model and non-linear model.

The log function conversion of uGLY with the constant "e" as the base was performed and used for the analysis.

Supplementary Table 16. Threshold effect analysis of Log_e_(uGLY) level and FLI stratified by gender after excluding outliers

| **Gender** | **Male** | **Female** |
| --- | --- | --- |
| Linear model |  |  |
| Beta coefficient (95%CI) | 1.40 (-0.70, 3.49) | 2.31 (0.14, 4.48) |
| Non-linear model |  |  |
| Inflection Point (K) | 0.08 | -0.99 |
| Beta coefficient (95%CI) (< K) | 1.95 (-0.52, 4.42) | 5.29 (0.94, 9.64) |
| Beta coefficient (95%CI) (> K) | -3.96 (-16.72, 8.81) | -0.49 (-4.65, 3.67) |
| LLR | 0.402 | 0.120 |

All covariates were adjusted in linear model and non-linear model.

The log function conversion of uGLY with the constant "e" as the base was performed and used for the analysis.

Supplementary Table 17. Threshold effect analysis of Log_e_(uGLY) level and FLI stratified by age after excluding outliers

| **Age (yrs)** | **< 40** | **40-60** | **> 60** |
| --- | --- | --- | --- |
| Linear model |  |  |  |
| Beta coefficient (95%CI) | 0.65 (-1.95, 3.26) | 3.41 (0.89, 5.93) | 2.18 (-0.53, 4.89) |
| Non-linear model |  |  |  |
| Inflection Point (K) | -1.32 | 0.19 | -0.6 |
| Beta coefficient (95%CI) (< K) | 6.74 (-1.08, 14.57) | 4.35 (1.47, 7.22) | 5.30 (0.97, 9.63) |
| Beta coefficient (95%CI) (> K) | -2.17 (-6.46, 2.13) | -10.70 (-31.81, 10.40) | -3.07 (-9.36, 3.22) |
| LLR | 0.103 | 0.183 | 0.068 |

All covariates were adjusted in linear model and non-linear model

The log function conversion of uGLY with the constant "e" as the base was performed and used for the analysis.

Supplementary Table 18. Threshold effect analysis of Log_e_(uGLY) level and FLI stratified by race/ethnicity after excluding outliers

| **Race/ethnicity** | **Mexican American** | **White** | **Black** | **Other Race** |
| --- | --- | --- | --- | --- |
| Linear model |  |  |  |  |
| Beta coefficient (95%CI) | 0.32 (-3.79, 4.43) | 1.61 (-0.75, 3.97) | 1.78 (-1.73, 5.28) | 5.50 (2.48, 8.52) |
| Non-linear model |  |  |  |  |
| Inflection Point (K) | -0.51 | -0.64 | -0.1 | -0.45 |
| Beta coefficient (95%CI) (< K) | -1.70 (-6.96, 3.55) | 3.49 (-0.28, 7.27) | -2.23 (-6.59, 2.14) | 8.99 (4.73, 13.24) |
| Beta coefficient (95%CI) (> K) | 9.34 (-5.81, 24.50) | -1.72 (-7.46, 4.01) | 26.73 (9.84, 43.62) | -6.46 (-17.21, 4.28) |
| LLR | 0.216 | 0.207 | 0.003 | 0.022 |

All covariates were adjusted in linear model and non-linear model

The log function conversion of uGLY with the constant "e" as the base was performed and used for the analysis.

Supplementary Table 19. Threshold effect analysis of Log_e_(uGLY) level and FLI stratified by hypertension after excluding outliers

| **Hypertension** | **Yes** | **No** |
| --- | --- | --- |
| Linear model |  |  |
| Beta coefficient (95%CI) | 0.83 (-1.71, 3.37) | 2.21 (0.36, 4.06) |
| Non-linear model |  |  |
| Inflection Point (K) | 0.17 | -1.27 |
| Beta coefficient (95%CI) (< K) | 1.89 (-1.01, 4.79) | 5.53 (0.23, 10.83) |
| Beta coefficient (95%CI) (> K) | -11.53 (-28.10, 5.04) | 0.58 (-2.48, 3.64) |
| LLR | 0.136 | 0.188 |

All covariates were adjusted in linear model and non-linear model.

The log function conversion of uGLY with the constant "e" as the base was performed and used for the analysis.

Supplementary Table 20. Threshold effect analysis of Log_e_(uGLY) level and FLI stratified by diabetes after excluding outliers

| **Diabetes** | **Yes** | **No** | **Borderline** |
| --- | --- | --- | --- |
| Linear model |  |  |  |
| Beta coefficient (95%CI) | 0.28 (-3.53, 4.09) | 2.08 (0.43, 3.74) | 4.85 (-5.75, 15.44) |
| Non-linear model |  |  |  |
| Inflection Point(K) | -1.55 | -0.38 | 0.1 |
| Beta coefficient (95%CI) (< K) | -24.09 (-45.72, -2.47) | 3.24 (0.96, 5.51) | 11.53 (-1.71, 24.76) |
| Beta coefficient (95%CI) (> K) | 3.95 (-1.01, 8.91) | -2.03 (-7.83, 3.77) | -58.12 (-135.86, 19.62) |
| LLR | 0.022 | 0.146 | 0.071 |

All covariates were adjusted in linear model and non-linear model.

The log function conversion of uGLY with the constant "e" as the base was performed and used for the analysis.
