# Supplementary material for: Analysis of the association between urinary glyphosate exposure and fatty liver index: a study for US adults
Source: BMC Public Health. 2024 Mar 5;24:703. doi: 10.1186/s12889-024-18189-3 (PMC10916137; doi:10.1186/s12889-024-18189-3)
Supplement: Supplementary file 2 — Supplementary Material 2 [file 12889_2024_18189_MOESM2_ESM.doc]

Supplementary figures:


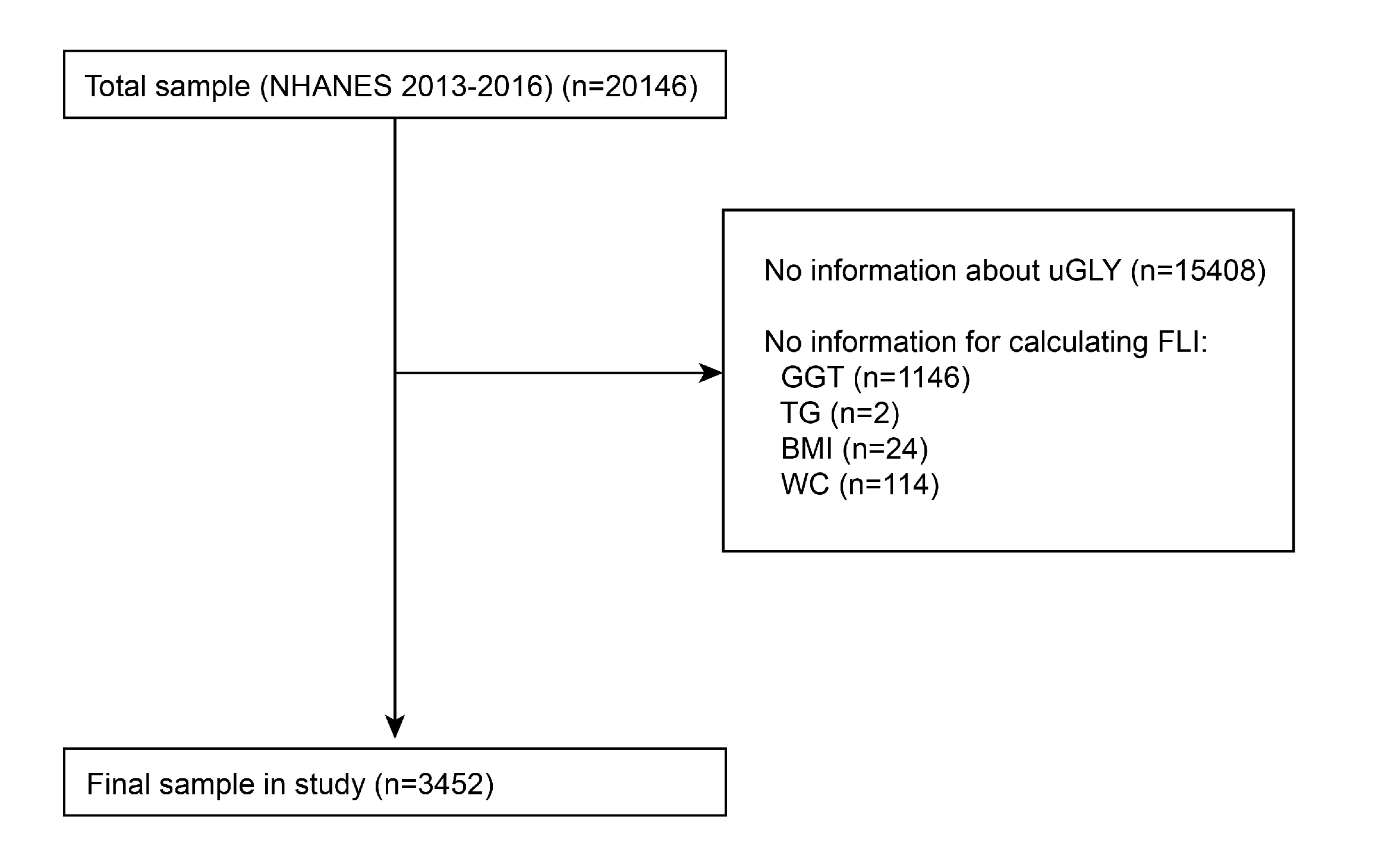


Supplementary figure 1. Flow chart for participants (includes participants younger than 20 years of age, presence of heavy alcohol consumption, use of medications that interfere with fat metabolism, viral hepatitis, substandard urine samples, and presence of renal weakness/failure).
